# Large cerebral tuberculoma

**DOI:** 10.1002/ccr3.8827

**Published:** 2024-04-26

**Authors:** Nor Osman Sidow

**Affiliations:** ^1^ Mogadishu Somalia Turkey Training and Research Hospital Mogadishu Somalia

**Keywords:** cerebral, seizure, tuberculoma, vasogenic edema

## Abstract

Here, we are presenting a young previous healthy child with seizures and right side hemiparesis for 6 months. After blood work and an MRI brain with IV contrast, it is confirmed that the child has large cerebral tuberculoma. The child is improved with TB treatment and surgery.

A previous healthy 7‐year‐old boy comes at the neurology outpatient department with right side hemiparesis, slurred of speech, and seizure for 6 months. No previous history of tuberculosis or other chronic diseases. Physical examination: he was conscious, alert, afebrile, and no meningeal signs, there was right side weakness (power 3/5), and motor aphasia. Magnetic resonance imaging of the brain with intravenous gadolinium contrast demonstrated large ring enhancing lesion in the left cerebral hemisphere. Widespread vasogenic edema was observed around this described lesion (Figure 1A). An 18‐mm left to right midline shift was observed with compression of the left lateral ventricle. In addition, leptomeningeal enhancement areas were detected in both cerebral hemispheres. Findings are highly compatible with intracranial tuberculoma and accompanying meningitis (Figure 1B,C), and the HIV test was negative. Left side hemi‐craniotomy with total resection was planned and performed. Histological findings showed caseous necrosis with giant multinucleated cell of acid‐fast bacilli. The patient was initiated on antituberculosis treatment for 1 year (Four regimens: rifampin, isoniazid, pyrazinamide, and ethambutol). His symptoms improved after 4 months of treatment with being able to walk unaided and with right side power of (4/5). Tuberculoma and tuberculosis (TB) meningitis are two of the devastating complications of TB, which require prolonged treatment and have a poor prognosis.^1^, ^2^, ^3^

**FIGURE 1:**
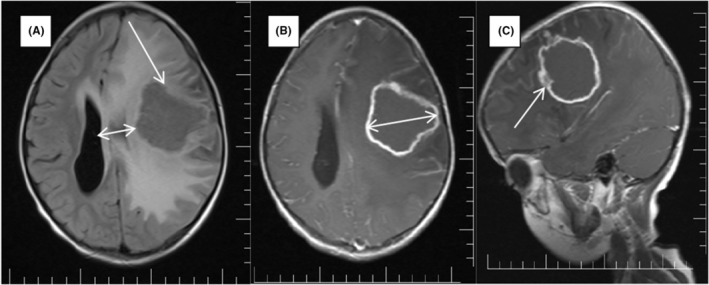
This is an MRI brain that shows a lesion with vasogenic edema in the right frontoparietal region (A), and after IV Gad contrast, it shows lesion with leptomeningeal enhancement of both cerebral hemispheres (B and C).

## AUTHOR CONTRIBUTIONS

**Nor Osman Sidow:** Conceptualization; writing – original draft; writing – review and editing.

## FUNDING INFORMATION

No funding is available.

## CONFLICT OF INTEREST STATEMENT

The author has no conflict of interest to report.

## CONSENT

A written informed consent was obtained for the patient's father to publish this image report.

## Data Availability

The data of this case image are available by the corresponding author if there is a need.

## References

[1] Wilkinson RJ , Rohlwink U , Misra UK , et al. Tuberculous meningitis. Nat Rev Neurol. 2017;13(10):581‐598. 28884751 10.1038/nrneurol.2017.120

[2] Marx GE , Chan ED . Tuberculous meningitis: diagnosis and treatment overview. Tuberc Res Treat. 2011;2011:1‐9. 10.1155/2011/798764 PMC3335590 22567269

[3] DeLance AR , Safaee M , Oh MC , et al. Tuberculoma of the central nervous system. J Clin Neurosci. 2013;20(10):1333‐1341. 23768968 10.1016/j.jocn.2013.01.008

